# A Case Report of Remitting Seronegative Symmetrical Synovitis With Pitting Edema Causing Severe Anemia, Acute Progressive Dementia, and Chronic Eczema in an Older Female

**DOI:** 10.7759/cureus.58940

**Published:** 2024-04-24

**Authors:** Taichi Matsubara, Chihiro Uda, Chiaki Sano, Ryuichi Ohta

**Affiliations:** 1 Family Medicine, Fuchu Hospital, Osaka, JPN; 2 Community Medicine Management, Shimane University Faculty of Medicine, Izumo, JPN; 3 Communiy Care, Unnan City Hospital, Unnan, JPN

**Keywords:** allergic rash, dementia, family medicine, general medicine, rural areas, older adult, rs3pe syndrome

## Abstract

Remitting seronegative symmetrical synovitis with pitting edema (RS3PE) syndrome is a type of seronegative arthritis characterized by a favorable prognosis (Remitting), absence of rheumatoid factor (Seronegative), symmetry (Symmetrical), and synovitis with pitting edema on the backs of the hands and feet. The cause of RS3PE syndrome remains unknown, but involvement of the immune system is suspected, and steroids are highly effective. Here, we present a case of an 86-year-old woman with severe anemia and bilateral lower limb edema accompanied by chronic eczema, considered to be caused by RS3PE syndrome. The patient's symptoms included bilateral lower limb edema, allergic rash, cognitive decline, and difficulty in moving, all of which were attributed to RS3PE syndrome. Given the variety of systemic symptoms associated with RS3PE syndrome, which can significantly impair the activities of daily living (ADLs) in the elderly, early detection and treatment are crucial.

## Introduction

Remitting seronegative symmetrical synovitis with pitting edema (RS3PE) syndrome is a type of seronegative arthritis more common in elderly males, named for its characteristics: (1) a favorable prognosis (Remitting), (2) negative for rheumatoid factor (Seronegative), (3) symmetrical in presentation (Symmetrical), and (4) synovitis with pitting edema on the dorsum of the hands and feet (Synovitis with Pitting Edema) [1]. The etiology is unknown, but there have been reports of approximately half of the cases being positive for the human leukocyte antigen (HLA)-B7, suggesting immune system involvement, and reports implicating HLA-CW7 and HLADQW2 [2]. Typical manifestations include sudden onset of symmetrical arthritis and synovitis of the fingers and hands, with pitting edema, though elbow, shoulder, knee, and foot involvement can also occur [3]. While no established diagnostic criteria exist, the diagnosis of RS3PE is considered when a patient exhibits (1) bilateral pitting edema, (2) acute onset polyarthritis, (3) onset at age 50 or older, and (4) negative rheumatoid factor. The disease typically responds well to relatively low doses of steroids (prednisone 10-15 mg/day) and tends to improve, with most cases eventually remitting [4]. Treatment is usually successful with steroids alone, allowing for tapering and discontinuation, though some cases are resistant, necessitating consideration of immunosuppressants like methotrexate (MTX). Notably, RS3PE syndrome has a high rate of cancer co-occurrence; screening for malignancy is also crucial [5].

We encountered a case of an octogenarian female presenting with subacute to chronic progression of bilateral lower extremity edema, difficulty in moving, chronic eczema, and cognitive decline, ultimately diagnosed with RS3PE syndrome. Although the clinical course was atypical, through exclusion diagnosis and responding to prednisolone treatment, symptoms improved until independence in activities of daily living (ADLs) was regained. This case highlights the pathophysiology of RS3PE syndrome in the elderly and its presentation as a syndrome.

## Case presentation

An 86-year-old female visited the Department of General Medicine of our hospital with complaints of forgetfulness, anemia, and bilateral lower leg edema. She had been visiting a dermatologist for a generalized eczema without a definitive diagnosis for several years, managing with moisturizers and experiencing periods of remission and exacerbation. Two months before her visit, she began to exhibit increased forgetfulness, and a month ago, she started experiencing urinary and fecal incontinence. Concurrently, the bilateral lower leg edema, which had been present for a few months, became more prominent, and her sleeping hours increased. Although her appetite was unchanged, the edema made meal preparation difficult, leading to decreased food intake over the past two weeks. She had been spending more time lying down during the day. As she could not live by herself, her family took her to the hospital. Her past medical history included bronchial asthma. She was not on any medications or topical treatments at the time of the visit, though she had been taking amlodipine 5 mg until two months before the visit.

**Figure 1:**
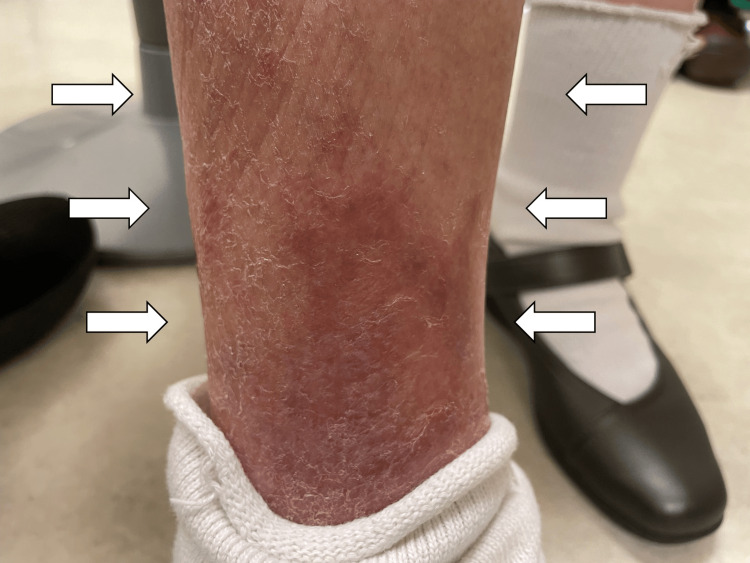
Exudative eczema and mild edema on right leg at first visit (white arrows)

At her initial visit, she was alert, with vital signs as follows: temperature 36.4 °C, blood pressure 133/67 mmHg, pulse rate 84 beats per minute, respiratory rate 16 breaths per minute, and oxygen saturation 96% in room air. She had exudative eczema primarily on both lower legs, mild edema on both upper limbs, and fast pitting edema on both lower legs (Figure 1).

There was no lymphadenopathy or eyelid edema, and heart and lung auscultation were normal. Blood tests showed moderate anemia and elevated inflammatory markers (Table 1).

**Table 1:** Initial laboratory data of the patient CRP, C-reactive protein; Ig, immunoglobulin

Paremeter	Level	Reference
White blood cells	8.8	3.5–9.1 × 10^3^/μL
Neutrophils	85.5	44.0–72.0%
Lymphocytes	7.4	18.0–59.0%
Hemoglobin	8.9	11.3–15.2 g/dL
Hematocrit	28.0	33.4–44.9%
Mean corpuscular volume	90.3	79.0–100.0 fl
Platelets	40.0	13.0–36.9 × 10^4^/μL
Erythrocyte sedimentation rate	20	2–10 mm/hour
Total protein	6.5	6.5–8.3 g/dL
Albumin	2.6	3.8–5.3 g/dL
Total bilirubin	6.3	0.2–1.2 mg/dL
Aspartate aminotransferase	15	8–38 IU/L
Alanine aminotransferase	8	4–43 IU/L
Lactate dehydrogenase	172	121–245 U/L
Blood urea nitrogen	15.8	8–20 mg/dL
Creatinine	0.48	0.40–1.10 mg/dL
Serum Na	138	135–150 mEq/L
Serum K	4.1	3.5–5.3 mEq/L
Serum Cl	101	98–110 mEq/L
Ferrum	68	54-200 μg/dL
Ferritin	231	14.4–303.7 ng/mL
CRP	7.02	<0.30 mg/dL
IgG	1643	870–1700 mg/dL
IgM	43	35–220 mg/dL
IgA	337	110–410 mg/dL
Urine test	-	-
Leukocyte	Negative	Negative
Protein	Negative	Negative
Blood	Negative	Negative

A head computed tomography (CT) was performed to investigate cognitive decline, showing no abnormality. An echocardiogram and a venous ultrasound of the lower limbs were conducted, detecting normal cardiac function without valvular abnormalities and venous thrombosis. A chest-abdomen-pelvis CT was performed to investigate the causes of both edema and anemia, but no tumor lesions were observed. The fecal occult blood test was negative as well. The score of the mini-mental state examination (MMSE) was 12.

Despite elevated C-reactive protein (CRP) levels at the initial visit, stability of vital signs, and specific physical findings for infection, an infection was not actively suspected. The anemia, with low transferrin saturation and elevated ferritin, suggested a mix of iron deficiency and inflammation. Zinc deficiency (54 μg/dL (reference, 80 to 130)) was also noted, prompting zinc of 75 mg daily and sodium ferric citrate of 50 mg daily.

**Figure 2:**
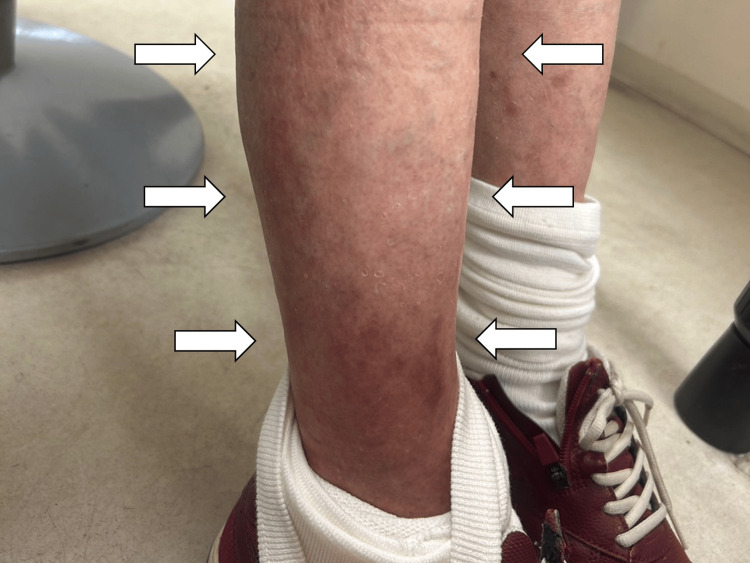
Right leg one month after the treatment (white arrows)

At a follow-up visit two weeks later, the edema in both upper arms and lower legs had worsened, and there was tenderness in both shoulder and hand joints. The anemia had also worsened (hemoglobin of 8.0 g/dL). No malignant tumors were found to cause the anemia. There were no intracranial findings to explain the cognitive decline. There was also no lower limb venous thrombosis, cardiac dysfunction, or renal dysfunction to account for the edema. Additional laboratory tests showed negative results for rheumatoid factor, anti-citrullinated protein antibodies, antinuclear antibodies, and anti-neutrophil cytoplasmic antibodies. There was a persistent positive inflammatory response without a significant increase in globulin and a mild increase in soluble interleukin (IL) two receptors. The presence of leukocytosis, elevated CRP, and increased erythrocyte sedimentation rate, along with clinical findings, led to the consideration of RS3PE syndrome with eosinophilia, and treatment with oral prednisolone 15mg/day was initiated. In the follow-up one month later, improvement in inflammation marker (CRP of 0.03 mg/dL), bilateral lower leg edema, and anemia (hemoglobin of 11.6 g/dL) were noted (Figure 2).

In the outpatient department, the prednisolone continued to be tapered gradually without any exacerbation. Her cognitive function recovered with a score of MMSE of 24, as she was living alone.

## Discussion

We experienced a case of RS3PE syndrome diagnosed from bilateral lower limb edema, rash, cognitive decline, and difficulty in moving that began with a subacute course and responded to PSL (prednisolone) treatment. In the elderly, Polymyalgia Rheumatica (PMR) and RS3PE syndrome are diseases that significantly reduce Activities of Daily Living (ADL), necessitating early diagnosis and treatment [6]. Given the specific symptoms and pathophysiology in the elderly, these conditions can present with non-musculoskeletal symptoms, potentially delaying diagnosis [7]. It is essential to consider the possibility of these diseases, promptly conduct differential diagnoses, and start PSL early.

RS3PE syndrome may trigger various immune responses, necessitating its inclusion in differential diagnoses. McGonagle proposed a disease distribution that can classify immune-mediated diseases into primarily autoimmune (AD), primarily autoinflammatory (AID), or mixed types [8]. ADs are characterized by abnormal adaptive immune system activation, whereas AIDs result from abnormal innate immune system activation without autoantibodies or autoreactive T cells. While the cause of RS3PE syndrome remains unclear, some reports suggest it may include elements of both AD and AID [8,9]. RS3PE syndrome is also classified as a Vascular Endothelial Growth Factor (VEGF)-related disease [1,10]. Patients with RS3PE syndrome have significantly elevated serum VEGF concentrations, a vasoactive agent stimulating angiogenesis and vascular permeability, leading to synovitis, synovial angiogenesis, and subcutaneous edema [10]. In blood tests, this case also showed significant increases in IgE, IL-2R, and neutrophils, suggesting features of both autoimmune and autoinflammatory diseases [11]. In this case, the allergic rash observed before the diagnosis may be attributable to RS3PE syndrome, with elevated IgE levels suggesting antigen-specific IgE production following antigen exposure and B-cell differentiation into plasma cells, possibly induced by cytokines promoting class switching in RS3PE syndrome [12]. However, RS3PE syndrome with eosinophilia is rarely reported, leaving room for further discussion.

The progression of edema in RS3PE syndrome can be gradual, differing in pain progression from PMR. The edema observed in this case, consistent with RS3PE syndrome caused by synovitis of the flexor and extensor tendon sheaths, does not contradict the presence of edema extending to the lower legs [13]. The progression of fatigue could not be denied as being symptomatic due to lower leg edema, but cachexia may have been a contributing factor [14]. In cachexia, the balance between pro-inflammatory and anti-inflammatory cytokines is crucial. Pro-inflammatory cytokines, including IL-1β, IL-6, tumor necrotizing factor (TNF)-α, and Interferon (IFN)-γ, cause appetite loss and increased energy expenditure [15]. At the same time, muscle atrophy involves IL-1β, IL-6, and TNF-α, likely leading to systemic fatigue as a symptom.

Cognitive decline and anemia, in this case, might be caused by chronic inflammation-induced intracranial inflammation and hematopoietic suppression. The cognitive decline progressed over months, becoming rapidly progressive dementia [16]. Causes of rapidly progressive dementia include neurodegenerative diseases, infectious diseases, metabolic disorders, autoimmune diseases, endocrine disorders, and tumor-related conditions [17]. In our case, blood counts, electrolytes, liver function tests, syphilis testing, autoantibodies, urine tests, and head CT were performed. Still, no evidence of infection, endocrine disorders, metabolic disorders, or intracranial organic diseases was found. In RS3PE syndrome and VEGF, inflammatory cytokines like IL-6 and Matrix metalloproteinase (MMP)-3 are produced, potentially causing chronic intracranial inflammation leading to ischemic changes [18]. In our case, the simultaneous onset of noticeable lower leg edema and rapid cognitive decline might indicate acceleration due to frailty, with prednisolone treatment improving cognitive function and supporting ischemic changes due to intracranial inflammation. The anemia was also considered to result from chronic inflammation, with serum iron, ferritin, and TIBC test values supporting this conclusion. As the possibility of malignancy can remain, RS3PE syndrome with anemia effectively treated by prednisolone should be followed meticulously by checking symptoms and screening for malignancy. The comprehensive approach to patients with multiple medical issues is vital for older patient care. General medicine should enhance the approach through continual professional development regarding autoimmune diseases in general hospitals [19,20].

## Conclusions

We encountered a case presenting with bilateral lower limb edema, difficulty moving, chronic eczema, and cognitive decline, progressing from a subacute to a chronic course, ultimately diagnosed with RS3PE syndrome. The edema, rash, fatigue, and cognitive decline observed in this case were inflammatory changes associated with RS3PE syndrome. The symptoms improved with prednisolone treatment, indicating the effectiveness of corticosteroids in managing inflammation. The wide range of systemic symptoms can significantly reduce ADL in the elderly, highlighting the importance of keeping this disease in mind for early diagnosis and treatment to prevent deterioration of quality of life.
